# El Tor Biotype *Vibrio cholerae* Activates the Caspase-11-Independent Canonical Nlrp3 and Pyrin Inflammasomes

**DOI:** 10.3389/fimmu.2019.02463

**Published:** 2019-10-29

**Authors:** Michail Mamantopoulos, Ulrika C. Frising, Tomoko Asaoka, Geert van Loo, Mohamed Lamkanfi, Andy Wullaert

**Affiliations:** ^1^Department of Internal Medicine and Pediatrics, Ghent University, Ghent, Belgium; ^2^VIB-UGent Center for Inflammation Research, VIB, Ghent, Belgium; ^3^Department of Biomedical Molecular Biology, Ghent University, Ghent, Belgium; ^4^Ghent Gut Inflammation Group (GGIG), Ghent University, Ghent, Belgium; ^5^Janssen Immunosciences, World Without Disease Accelerator, Pharmaceutical Companies of Johnson & Johnson, Beerse, Belgium

**Keywords:** *Vibrio cholerae* El Tor biotype, caspase-1, caspase-11, Nlrp3, pyrin

## Abstract

*Vibrio cholerae* is a Gram-negative enteropathogen causing potentially life-threatening cholera disease outbreaks, for which the World Health Organization currently registers 2–4 million cases and ~100.000 cholera-associated deaths annually worldwide. Genomic *Vibrio cholerae* research revealed that the strains causing this ongoing cholera pandemic are members of the El Tor biotype, which fully replaced the Classical biotype that caused former cholera pandemics. While both of these biotypes express the characteristic Cholera Toxin (CT), the El Tor biotype additionally expresses the accessory toxins hemolysin (hlyA) and multifunctional auto-processing repeat-in-toxin (MARTX). Previous studies demonstrated that the Classical biotype of *Vibrio cholerae* triggers caspase-11-dependent non-canonical inflammasome activation in macrophages following CT-mediated cytosolic delivery of LPS. In contrast to the Classical biotype, we here show that El Tor *Vibrio cholerae* induces IL-1β maturation and secretion in a caspase-11- and CT-independent manner. Instead, we show that El Tor *Vibrio cholerae* engages the canonical Nlrp3 inflammasome for IL-1β secretion through its accessory hlyA toxin. We further reveal the capacity of this enteropathogen to engage the canonical Pyrin inflammasome as an accessory mechanism for IL-1β secretion in conditions when the pro-inflammatory hlyA-Nlrp3 axis is blocked. Thus, we show that the *V. cholerae* El Tor biotype does not trigger caspase-11 activation, but instead triggers parallel Nlrp3- and Pyrin-dependent pathways toward canonical inflammasome activation to induce IL-1β-mediated inflammatory responses. These findings further unravel the complex inflammasome activating mechanisms that can be triggered when macrophages face the full arsenal of El Tor *Vibrio cholerae* toxins, and as such increase our understanding of host-pathogen interactions in the context of the *Vibrio cholerae* biotype associated with the ongoing cholera pandemic.

## Introduction

*Vibrio cholerae* is a Gram-negative enteropathogen that caused numerous cholera outbreaks in the past and remains a public health threat also today, as illustrated by the current cholera epidemic in Yemen that is among the largest outbreaks in the last century (1). A recent genomic characterization of *V. cholerae* strains collected in Yemen throughout 2016 and 2017 revealed that all of these strains belonged to the El Tor biotype (1, 2). Similar El Tor driven epidemics have affected regions in Africa and Latin America in the past decades (3). Indeed, the El Tor biotype is responsible for the ongoing seventh *V. cholerae* pandemic for which the WHO registers 2–4 million cases and ~100.000 cholera-associated deaths annually worldwide, and has completely replaced the Classical biotype that caused former pandemics (4, 5).

*V. cholerae* carries an arsenal of toxins contributing to infection. Cholera toxin (CT) is the virulence factor responsible for triggering the diarrheal manifestations of cholera disease. CT accomplishes this through its A subunit (CTA) that activates the adenylate cyclase in intestinal epithelial cells, which results in a net secretion of chloride ions and water into the intestinal lumen (6). While the molecular mechanisms by which they contribute to cholera pathology is less clear, the accessory toxins hemolysin (hlyA) and multifunctional auto-processing repeat-in-toxin (MARTX) are characterized by their pore-forming and cytoskeleton-disrupting cellular effects, respectively (7, 8). Within the toxigenic *V. cholerae* O1 serogroup that caused all seven cholera pandemics thus far, the Classical and El Tor biotypes can be distinguished based on the absence or the presence, respectively, of both hlyA and MARTX (9–14). However, compared to the Classical biotype, it is not clear how the additional expression of the accessory hlyA and MARTX toxins alters host innate immune responses to the *V. cholerae* El Tor biotype.

Inflammasomes represent a family of signaling complexes in which detection of specific bacterial components or cellular danger signals through various cytosolic receptors leads to activation of the catalytic caspase-1 component that mediates maturation and secretion of the Interleukin (IL)-1β and IL-18 pro-inflammatory cytokines (15). For instance, both Nlrc4 and Nlrp3 inflammasomes contribute to caspase-1 activation upon infection with *Salmonella* Typhimurium (16–18). In contrast, *Clostridium difficile* uses its cytotoxins to inactivate host Rho GTPases, which in turn triggers the Pyrin inflammasome to activate caspase-1 (19). The above examples constitute so-called canonical inflammasome pathways that rely solely on caspase-1 activity to mediate their innate immunity effects. However, several Gram-negative enteropathogens such as *Citrobacter rodentium* and *Escherichia coli* were shown to trigger a non-canonical inflammasome pathway that requires additional caspase-11 activity (20, 21). The non-canonical inflammasome pathway is initiated upon recognition of cytosolic Lipopolysaccharide (LPS) by caspase-11, which triggers inflammasome activation in an Nlrp3-dependent manner (22). Akin to this mechanism, the non-enzymatic CT subunit B (CTB) of *V. cholerae* binds to GM_1_ gangliosides on the host cellular surface and as such can act as a carrier that facilitates cytosolic entry of LPS (20). Consistent with the mechanism of non-canonical inflammasome activation, challenging macrophages with a combination of CTB and LPS, as well as with live *V. cholerae*, indeed resulted in a caspase-11- and Nlrp3-dependent manner of IL-1β secretion (20, 21). However, this non-canonical pathway of inflammasome activation upon *V. cholerae* infection was established using the Classical biotype of the bacterium (20). Given that this biotype harbors deletions in the genes encoding hlyA and MARTX (9–14), it remains to be elucidated whether and how the toxins expressed by the El Tor biotype impact on the mode of *V. cholerae* induced inflammasome activation.

In contrast to reports based on the Classical biotype, we here show that the CT- and caspase-11-dependent non-canonical pathway is redundant for triggering inflammasome responses upon infection with El Tor *V. cholerae*. Instead, the latter biotype predominantly activates the canonical Nlrp3 inflammasome through its hlyA toxin. Intriguingly, in conditions of impaired Nlrp3 inflammasome activation, El Tor *V. cholerae* initiated a backup pathway toward IL-1β maturation and secretion by triggering the canonical Pyrin inflammasome in a CT- and hlyA-independent manner. Altogether, our study shows that host inflammasome responses to the El Tor biotype *V. cholerae* are independent of CT, but instead are triggered by concerted actions of other toxins that activate distinct canonical Nlrp3 and Pyrin inflammasomes.

## Materials and Methods

### Ethics Statement

All animal experiments were performed according to institutionally approved protocols according to national (Belgian Laws 14/08/1986 and 22/12/2003, Belgian Royal Decree 06/04/2010) and European (EU Directives 2010/63/EU, 86/609/EEG) animal regulations. Animal protocols were reviewed and approved by the Ethical Committee Animal Experimentation—Ghent University—Faculty of Medicine and Health Sciences (permit number LA1400536) with approval ID ECD 14/40. All necessary efforts were made to minimize suffering of the animals.

### Mice

The caspase1/11^−/−^ (23), caspase11^−/−^ (24), ASC^−/−^ (25), Nlrp3^−/−^ (24), Nlrc4^−/−^ (25), Aim2^−/−^ (26), and Pyrin^−/−^ (27) mice used in this study, either generated on C57BL/6 background or backcrossed at least ten generations to C57BL/6J background, have been described previously. Wild-type (WT) C57BL/6J mice were originally obtained from Charles River but were—as all mice used in this study—bred and housed in individually ventilated cages (IVC) in the Specific Pathogen Free facility at Ghent University in a 12-h light-12-h dark cycle and were fed autoclaved standard rodent feed (Ssniff, Soest, Germany) at libitum with free access to drinking water.

### Bacterial Strains

The *V. cholerae* El Tor N16961 strain, originally isolated from a cholera patient in Bangladesh, was obtained from Pasteur Institute, Paris, France. The isogenic JBK70 (Δ*ctxAB*) and CvD104 (Δ*ctxAB/hlyA*) mutants of this *V. cholerae* El Tor N16961 strain were generated and kindly provided by James B. Kaper (University of Maryland, Baltimore, MD) (28). All the bacterial strains used for this study were grown in Luria-Bertani (LB) broth at 37°C while shaking. When indicated, El Tor *V. cholerae* strains were grown under AKI conditions for boosting CT production as described (29, 30).

### Cholera Toxin Detection

Secretion of Cholera Toxin in culture medium was detected using a GM_1_-ELISA method. In short, microwell plates were coated with GM_1_ ganglioside (Sigma Aldrich) overnight at RT. Plates were washed with 0.05% Tween 20 in PBS buffer and blocked with 1% BSA in PBS for 30 min at RT. Supernatants from *V. cholerae* cultures grown to an identical optical density (OD) for ensuring equal numbers of bacteria were added in duplicate (100 μl/well) and incubated for 1 h at 37°C. For a standard curve, selected wells were supplied with purified Cholera Toxin (Enzo life sciences) in PBS with 0.1% BSA in a concentration range from 0.1 to 20 ng/ml. Immobilized CT was detected with 100 μl/well Anti-Cholera Toxin subunit B goat primary antibody (Merck Millipore), followed by rabbit anti-goat horseradish peroxidase conjugate (Jackson ImmunoResearch). Results were visualized upon addition of p-nitrophenyl phosphate buffer (Sigma-Aldrich) for conjugate activation and the resulting OD at 405 nm was measured by spectrophotometry.

### *V. cholerae* Infection of Bone Marrow-Derived Macrophages (BMDMs)

Murine bone marrow cells were differentiated in Iscove's Modified Dulbecco's Medium (IMDM) supplemented with 30% L929 cell conditioned medium, 10% heat-inactivated fetal bovine serum (FBS), 1% non-essential amino acids and 1% penicillin-streptomycin. The cells were incubated for 6 days at 37°C and 5% CO_2_. BMDMs were seeded and incubated overnight at 37°C. Next BMDMs were primed with 500 ng/ml ultrapure LPS from *Salmonella minnesota* (InvivoGen) for 3 h. Subsequently, the LPS-containing medium was aspirated and the BMDMs were infected with *V. cholerae* at multiplicity of infection (MOI) 50 in Iscove's Modified Dulbecco's Medium (IMDM) supplemented with 10% fetal bovine serum (FBS) and 1% non-essential amino acids. When indicated, cells were additionally pre-treated with 10 μM of MCC950 (Sigma) for 30 min before infection. After infection, plates were centrifuged at 600xg for 10 min to ensure synchronization of the stage of infection. After incubating for 2 h at 37°C and 5% CO_2_, gentamicin (50 μg/ml) was added to kill remaining extracellular bacteria, after which the cells were incubated at 37°C and 5% CO_2_ for 18 h. At 24 h post infection cell culture supernatants were collected and used for cytokine analyses while supernatant containing cell lysates were used for Western blotting analysis.

### Cytokine Measurements

IL-1β and TNF cytokine secretion levels were analyzed from supernatants of cultured BMDMs by magnetic bead-based multiplex ELISA using the Bio-Rad Luminex technology according to the manufacturer's protocol. Data representation and analysis was performed in GraphPad Prism 6.0 software.

### Immunoblotting

Cells and culture supernatants were incubated in cell lysis buffer (20 mM Tris HCl (pH 7.4), 200 mM NaCl, 1% Nonidet P-40) for 10 min on ice followed by denaturation by boiling in Laemmli buffer for 10 min. Protein samples were resolved by SDS-PAGE electrophoresis and then transferred to polyvinylidene fluoride (PVDF) membranes by semi-dry blotting. The PVDF membrane blocking, antibody incubation, and washing steps were performed using PBS containing 0.05% Tween 20 (v/v) together with 3% (w/v) non-fat dry milk. The incubation of the membranes with primary antibody was performed overnight at 4°C and the primary antibodies used in this study included: caspase-1 (AG-20B-0042-C100, 1:1,000, Adipogen), IL-1β (GTX74034, 1:3,000, GeneTex) and γ-Tubulin (T6557-100UL, 1:1,000, Sigma Aldrich). HRP-conjugated secondary anti-mouse and anti-rabbit antibodies (111-035-144 and 115-035-146; 1:5,000, Jackson ImmunoResearch Laboratories), followed by ECL (Thermo Scientific) incubation were used for signal detection and visualization.

### Lactate Dehydrogenase (LDH) Assay

Lytic cell death was evaluated by detection of LDH released from cultured BMDMs in the cell culture supernatant by LDH assay (Promega) according to manufacturer's instructions. The data was plotted as percentage of total cell death using GraphPad Prism 7 software.

### Microscopy

BMDMs were infected with indicated *V. cholerae* genotypes at MOI50 and incubated for 2 h prior visualization with bright field microscopy. Images were taken at 400 × magnification.

## Results

### El Tor *V. cholerae* Activates the Caspase-11 Independent Canonical Pathway of Inflammasome Activation

**Figure 1:**
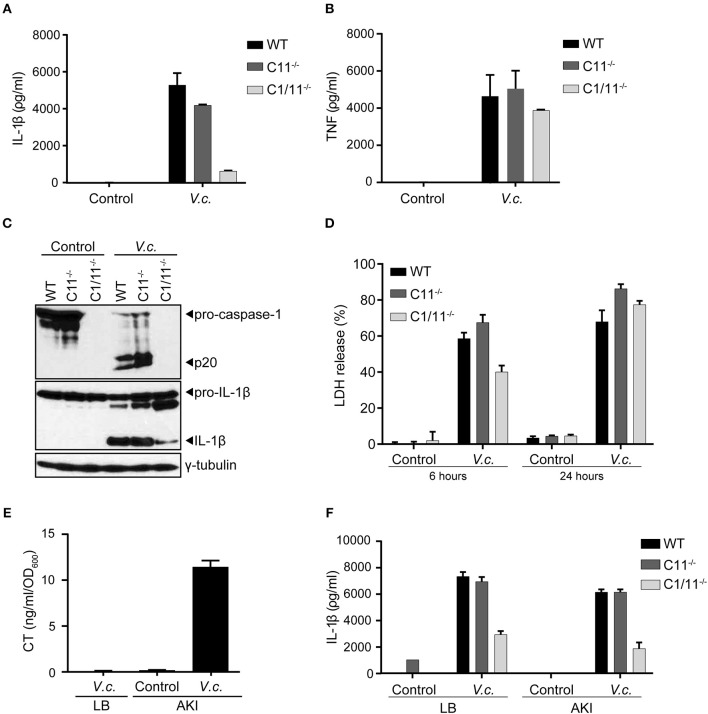
Caspase-11 independent canonical inflammasome activation upon El Tor *V. cholerae* infection. **(A–D)** LPS-primed BMDMs from WT, caspase-11^−/−^ and caspase-1/11^−/−^ mice were untreated (control) or infected with *V. cholerae* (*V.c*.) at MOI50. At 24 h post infection **(A)** cell culture supernatants were analyzed for secreted IL-1β and **(B)** TNF by ELISA, and **(C)** cell lysates were immunoblotted for IL-1β and caspase-1 maturation. **(D)** Cell death was determined 6 and 24 h post infection by LDH release assay. **(E)** ELISA for CT expression in *V. cholerae* culture supernatants cultured with LB or under AKI growth conditions. **(F)** LPS-primed BMDMs from WT, caspase-11^−/−^ and caspase-1/11^−/−^ mice were untreated (control) or infected with *V. cholerae* at MOI50 grown under indicated LB or AKI culture conditions. At 24 h post infection culture supernatants were analyzed for secreted IL-1β by ELISA. Data shown in **(A,B,D)** are means ± SD of triplicate wells from a representative experiment out of three independent experiments. Data shown in **(C)** are representative for three independent experiments. Data shown in **(E,F)** are means ± SD of triplicate wells from a single experiment.

The Classical biotype of *V. cholerae* was reported to activate the non-canonical inflammasome pathway that was dependent on the presence of caspase-11 (20). In order to characterize the mode of inflammasome activation triggered by the El Tor *V. cholerae* biotype, primary bone marrow-derived macrophages (BMDMs) from caspase-1/11^−/−^ or caspase-11^−/−^ mice were infected with the *V. cholerae* El Tor biotype N16961 strain (hereafter referred to as *V. cholerae*). In contrast to WT macrophages that secreted large amounts of IL-1β in the cell culture supernatant upon *V. cholerae* infection, caspase-1/11^−/−^ BMDMs displayed only little IL-1β secretion (Figure 1A). As the inflammasome-independent cytokine TNF was secreted normally from *V. cholerae* infected caspase-1/11^−/−^ cells (Figure 1B), this observation confirmed that *V. cholerae* infection induces a specific inflammasome-driven IL-1β response in primary macrophages. Surprisingly, *V. cholerae* infection induced similar IL-1β secretion levels in caspase-11-deficient cells compared to WT cells (Figure 1A). Moreover, western blotting analysis showed decreased processing of pro-IL-1β to its mature IL-1β form in caspase-1/11^−/−^ but not in caspase-11^−/−^ macrophages upon *V. cholerae* infection (Figure 1C). In addition, proteolytic caspase-1 processing upon *V. cholerae* infection was intact in the absence of caspase-11 (Figure 1C). Finally, we evaluated lytic cell death by measuring the levels of intracellular LDH released into the culture medium at different time points post infection. Notably, while at 6 h post infection cytotoxicity was slightly reduced in caspase-1/11^−/−^ but not in caspase-11^−/−^ macrophages, *V. cholerae* infection was equally cytotoxic to all genotypes at 24 h post infection (Figure 1D). This delay in cytotoxicity in caspase-1/11^−/−^ but not in caspase-11^−/−^ macrophages indicated a direct link between caspase-1-dependent (but caspase-11-independent) inflammasome activity and lytic cell death during early stages of infection, whereas additional inflammasome-independent pathways drive lytic cell death during later stages of *V. cholerae* infection. Together, these observations indicated that the El Tor *V. cholerae* biotype drives the caspase-1 dependent canonical pathway of inflammasome activation, rather than the non-canonical caspase-11 dependent pathway.

Because El Tor *V. cholerae* are known for their moderate CT production, we next aimed to boost CT expression in order to firmly establish the potential contribution of CT-mediated caspase-11 activation in inflammasome responses triggered by this biotype. Indeed, when cultured in LB medium under standard aerobic growth conditions, the N16961 El Tor strain did not secrete detectable levels of CT in the culture medium (Figure 1E), raising the possibility that the observed caspase-11 redundancy in inflammasome activation could stem from insufficient CT expression levels. We therefore cultured the N16961 El Tor strain in specific micro-aerobic growth conditions using AKI medium (29), which induced significant CT secretion in the medium (Figure 1E). Nevertheless, using this El Tor *V. cholerae* strain grown under these CT-boosting culturing conditions, we obtained similar results showing activation of the caspase-11-independent canonical inflammasome pathway. Indeed, IL-1β secretion was reduced in caspase-1/11^−/−^ but not in caspase-11^−/−^ macrophages infected by *V. cholerae* grown in AKI conditions (Figure 1F). As such, our results demonstrated that El Tor *V. cholerae* engages the canonical caspase-1-dependent but caspase-11-independent pathway of inflammasome activation leading to maturation and secretion of IL-1β.

### Hemolysin Is the Main Driver of Canonical Inflammasome Activation Upon El Tor *V. cholerae* Infection

**Figure 2:**
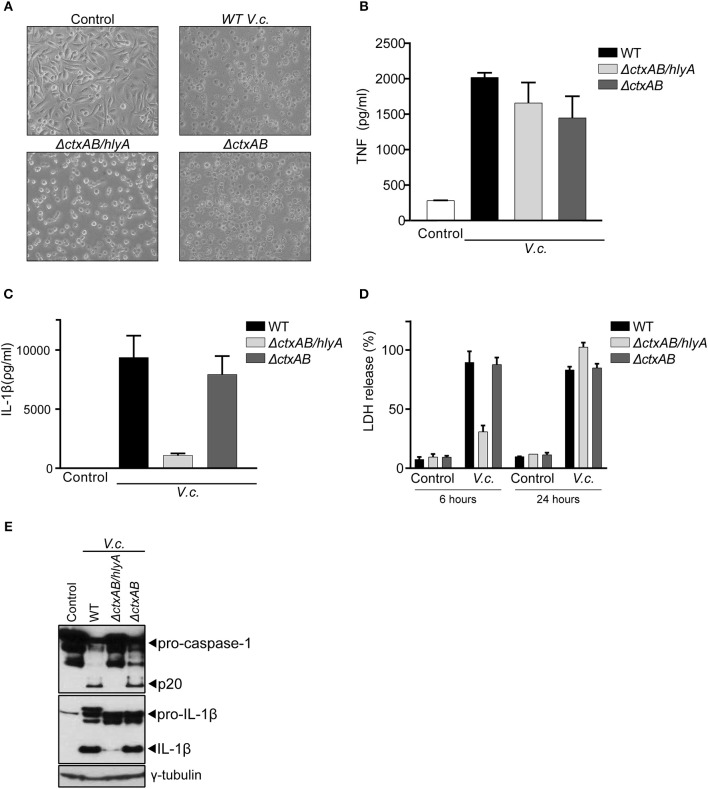
Hemolysin is the main trigger for canonical inflammasome activation upon El Tor *V. cholerae* infection. **(A)** Bright field images of WT BMDMs infected with different El Tor *V. cholerae* genotypes. **(B–E)** LPS-primed WT BMDMs were untreated (control) or infected with the indicated genotypes of *V. cholerae* (*V.c*.) at MOI50. At 24 h post infection **(B)** cell culture supernatants were analyzed for secreted TNF and **(C)** IL-1β by ELISA **(D)** cell death was determined 6 and 24 h post infection by LDH release assay. **(E)** Cell lysates at 24 h post infection were immunoblotted for IL-1β and caspase-1 maturation. Data shown in **(B–D)** are the means ± SD of triplicate wells from a representative experiment out of three independent experiments. Data shown in **(E)** are representative for three independent experiments.

Our above observations showed that caspase-11 did not contribute to IL-1β secretion upon infection with El Tor *V. cholerae*, suggesting that CT-mediated cytosolic entry of LPS does not play a role in inflammasome activation by this biotype. Therefore, we next used bacterial mutants to examine more directly the role of CT in *V. cholerae* induced IL-1β secretion, as well as to investigate which other toxins of this enteropathogen triggered the canonical inflammasome pathway. For this purpose, an El Tor *V. cholerae* N16961 strain isogenic mutant deleted for the genes encoding CTA and CTB (Δ*ctxAB*) was used, as well as this Δ*ctxAB* mutant harboring an additional deletion of the hemolysin A gene (Δ*ctxAB/hlyA*) (28). Upon challenging WT BMDMs with these different *V. cholerae* mutants, the characteristic rounding phenotype of macrophages was observed upon infection with all bacterial genotypes alike, confirming successful infection (Figure 2A). Additionally, similar levels of secreted TNF upon infection with the respective mutants (Figure 2B) provided further proof for equal macrophage infectivity of these different *V. cholerae* strains. Interestingly, the *V. cholerae* Δ*ctxAB* mutant triggered similar levels of IL-1β release as the WT pathogen (Figure 2C), thereby confirming the redundancy of CT-mediated caspase-11 activation in inflammasome responses to this bacterium. In contrast, the double Δ*ctxAB/hlyA* mutant displayed a clear reduction in IL-1β secretion compared to WT bacteria (Figure 2C), indicating a central role for hemolysin during canonical inflammasome activation in El Tor *V. cholerae* infected BMDMs. Moreover, analysis of macrophage cell death upon infection with the different *V. cholerae* genotypes revealed a hemolysin dependency at an early but not at a late time point of infection (Figure 2D). Due to the similarity with the aforementioned caspase-1-dependent cell death kinetics upon *V. cholerae* infection, this observation suggested that during early stages of infection El Tor *V. cholerae* hemolysin drives canonical inflammasome-dependent macrophage cell death. Consistently, western blotting analysis of caspase-1 and IL-1β maturation further validated El Tor hemolysin as the main driver of canonical inflammasome activation (Figure 2E). Altogether, although some residual proteolytic maturation of IL-1β could be observed in Δ*ctxAB/hlyA* infected cells (Figure 2E), thus pointing to additional inflammasome activating mechanisms in the absence of CT and hemolysin, our data indicated that El Tor *V. cholerae* activated the canonical inflammasome pathway predominantly through hemolysin.

### El Tor *V. cholerae* Predominantly Activates the Canonical Nlrp3 Inflammasome

**Figure 3:**
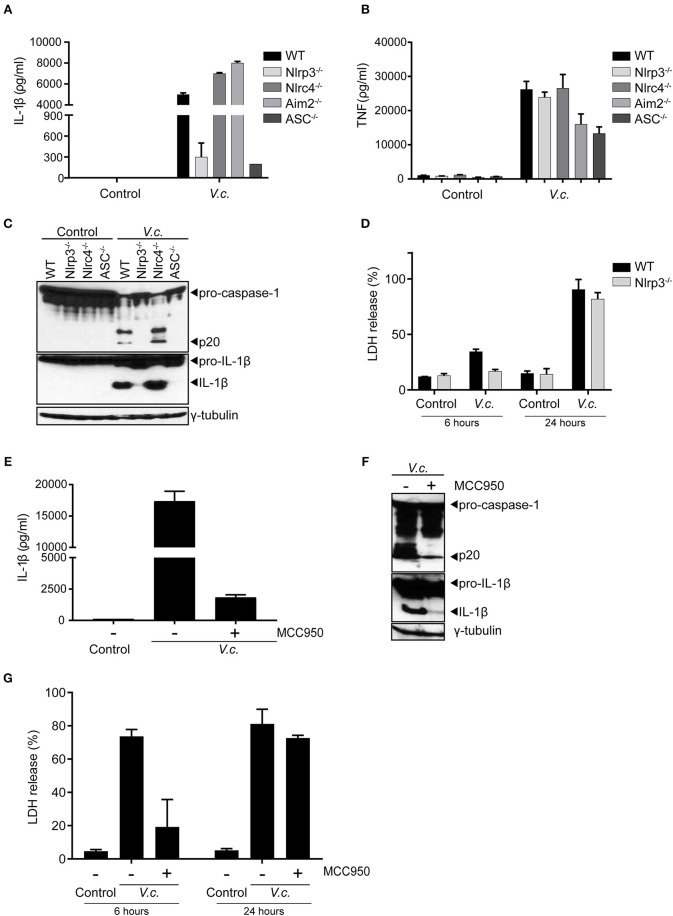
Maturation and secretion of IL-1β upon El Tor *V. cholerae* infection depends mainly on the Nlrp3 inflammasome. **(A–D)** LPS-primed BMDMs from mice with indicated genotypes were untreated (control) or infected with *V. cholerae* (*V.c*.) at MOI50. At 24 h post infection **(A)** cell culture supernatants were analyzed for secreted IL-1β and **(B)** TNF by ELISA, and **(C)** cell lysates of indicated BMDM genotypes were immunoblotted for IL-1β and caspase-1 maturation. **(D)** Cell death was determined at 6 and 24 h post infection by LDH release assay. **(E–G)** LPS-primed BMDMs from WT mice were untreated or pre-treated with 10 μM MCC950, as indicated, and then untreated (control) or infected with *V. cholerae* (*V.c*.) at MOI50. At 24 h post infection **(E)** cell culture supernatants were analyzed for secreted IL-1β by ELISA, and **(F)** cell lysates of indicated BMDM genotypes were immunoblotted for IL-1β and caspase-1 maturation. **(G)** Cell death was determined at 6 and 24 h post infection by LDH release assay. Data shown in **(A,B,D,E,G)** are the means ± SD of triplicate wells from a representative experiment out of three independent experiments. Data shown in **(C,F)** are representative for three independent experiments.

To further characterize the nature of canonical inflammasome activation triggered by El Tor *V. cholerae* infection, we next infected BMDMs derived from mice that were either deficient for the general inflammasome adaptor protein ASC or deficient for different inflammasome triggering receptors. As expected, since ASC is essential for IL-1β secretion downstream of all known inflammasomes, deletion of ASC disabled *V. cholerae* to induce IL-1β secretion from macrophages (Figure 3A). However, comparing IL-1β secretion from *V. cholerae* infected WT, Nlrp3^−/−^, Nlrc4^−/−^, and Aim2^−/−^ BMDMs revealed a specific role for the Nlrp3 inflammasome in this response. Indeed, only Nlrp3^−/−^ macrophages displayed a reduction in IL-1β secretion upon *V. cholerae* infection (Figure 3A), while the inflammasome-independent cytokine TNF was induced normally in the absence of Nlrp3 (Figure 3B). These central roles for both ASC and Nlrp3 in inflammasome responses upon El Tor infection were further confirmed by western blotting analysis showing decreased processing of both caspase-1 and IL-1β in the absence of these proteins (Figure 3C). In addition, LDH analysis revealed an Nlrp3-dependent delay in cell death of BMDMs infected with *V. cholerae*, again indicating that inflammasome-driven cell death is taking place mainly at the early time points post *V. cholerae* infection (Figure 3D). Finally, we used the specific Nlrp3 inflammasome inhibitor MCC950 (31) to address whether *V. cholerae* induced inflammasome responses could be targeted in a pharmacological manner. Indeed, treatment of WT macrophages with MCC950 decreased inflammasome-dependent IL-1β secretion upon El Tor infection (Figure 3E). In addition, MCC950 diminished *V. cholerae* induced processing of caspase-1 and IL-1β (Figure 3F) and it delayed lytic cell death in macrophages infected with *V. cholerae* (Figure 3G). Together with our prior observation that hlyA is the major El Tor toxin triggering inflammasome responses, these genetic and pharmacological experiments demonstrating that the El Tor *V. cholerae* biotype activates the canonical Nlrp3/ASC inflammasome suggest that hemolysin expressed by this pathogen triggers canonical inflammasome activation via the Nlrp3/ASC axis.

### El Tor *Vibrio cholerae* Activates the Pyrin Inflammasome in the Absence of Hemolysin-Dependent Nlrp3 Activation

**Figure 4:**
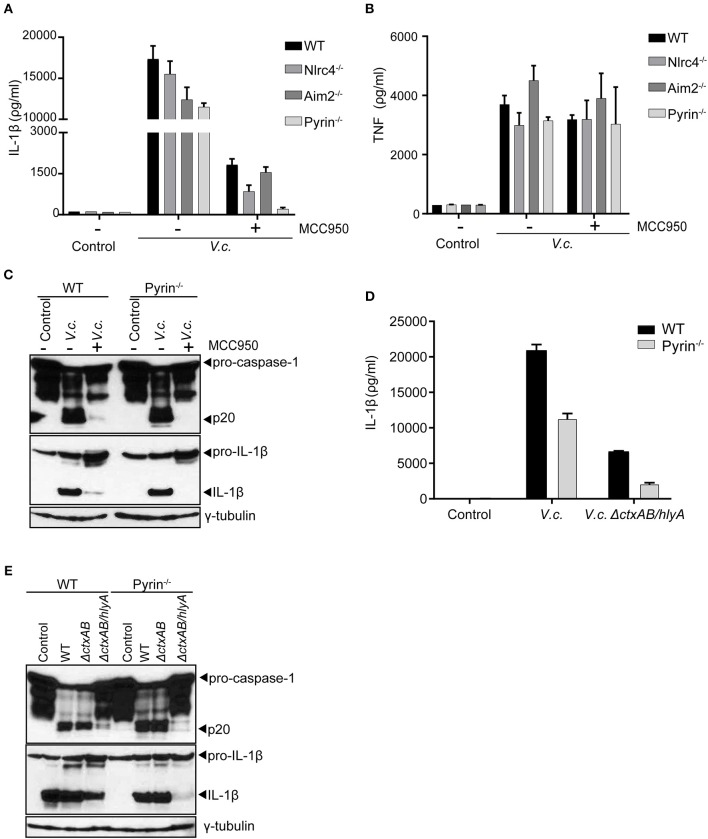
El Tor *V. cholerae* infection activates the Pyrin inflammasome in an Nlrp3- and hlyA-independent manner. **(A–C)** LPS-primed BMDMs from mice with indicated genotypes were left untreated or pre-treated with 10 μM MCC950 to block Nlrp3 activation, as indicated, and then untreated (control) or infected with *V. cholerae* (*V.c*.) at MOI50. At 24 h post infection **(A)** Cell culture supernatants were analyzed for secreted IL-1β and **(B)** TNF by ELISA, and **(C)** cell lysates of WT and Pyrin^−/−^ BMDMs were immunoblotted for IL-1β and caspase-1 maturation. **(D,E)** LPS-primed BMDMs from WT and Pyrin^−/−^ mice were untreated (control) or infected with *V. cholerae* (*V.c*.) with indicated genotypes at MOI50. At 24 h post infection **(D)** Cell culture supernatants were analyzed for secreted IL-1β by ELISA, and **(E)** cell lysates were immunoblotted for IL-1β and caspase-1 maturation. Data shown in **(A,B,D)** are the means ± SD of triplicate wells **(A,B)** from a single experiment, or **(D)** from a representative out of three independent experiments. Data shown in **(C,E)** are representative for three independent experiments.

Our above experiments collectively indicated that hemolysin-driven Nlrp3 activation was a dominant contributor to IL-1β processing upon El Tor *V. cholerae* infection. However, in the absence of bacterial hlyA (Figure 2E) as well as upon blocking host Nlrp3 activation (Figures 3C,F) we were able to detect residual IL-1β maturation. Therefore, we speculated that El Tor *V. cholerae* was capable of activating an additional hlyA/Nlrp3-independent inflammasome. To identify this putative Nlrp3-independent inflammasome, we took advantage of the MCC950 inhibitor to mitigate the dominant Nlrp3 effects and as such to investigate the roles of other inflammasomes. Consistent with our previous observations, MCC950-treated WT macrophages secreted residual amounts of IL-1β upon *V. cholerae* infection (Figure 4A). Interestingly however, while also MCC950-treated Nlrc4- and Aim2-deficient macrophages still released IL-1β in the culture medium upon El Tor *V. cholerae* infection, this IL-1β secretion was abolished in similarly treated Pyrin^−/−^ macrophages (Figure 4A). In contrast, TNF was secreted normally from MCC950-treated *V. cholerae* infected Pyrin^−/−^ macrophages (Figure 4B), demonstrating a still ongoing innate immune response to the bacterium. Moreover, in the setting of Nlrp3 inhibition by MCC950, western blotting analysis showed residual caspase-1 and IL-1β maturation in WT but not in Pyrin^−/−^ macrophages upon El Tor *V. cholerae* infection (Figure 4C). These observations indicated that El Tor *V. cholerae* can activate the Pyrin inflammasome as a backup mechanism in conditions of impaired Nlrp3 activation.

As a complementary approach to the above host genetic experiments, we next used the *V. cholerae* mutant strains to test whether the Pyrin inflammasome was also responsible for the residual inflammasome responses observed in the absence of both CT and hlyA. For this purpose, WT and Pyrin^−/−^ macrophages were infected with either WT *V. cholerae* or with the Δ*ctxAB/hlyA* mutant strain. Pyrin deficiency as such only moderately reduced IL-1β secretion in WT *V. cholerae* infected cells (Figure 4D), confirming that the Nlrp3 inflammasome is dominant in mediating this response. However, while the Δ*ctxAB/hlyA* mutant *V. cholerae* that cannot activate Nlrp3 remained capable of inducing low levels of IL-1β secretion from WT macrophages, Pyrin-deficient cells did not display this Δ*ctxAB/hlyA*-induced inflammasome response (Figure 4D). Moreover, western blotting analysis showed that the residual levels of processed caspase-1 and IL-1β as observed in Δ*ctxAB/hlyA* infected WT macrophages were diminished in Pyrin^−/−^ cells (Figure 4E). Together, while caspase-11-independent activation of the canonical Nlrp3 inflammasome by hemolysin appears the dominant mechanism toward IL-1β secretion, our observations indicated that the El Tor *V. cholerae* biotype can also activate the Pyrin inflammasome in the absence of either host Nlrp3 or bacterial hemolysin expression.

## Discussion

El Tor *V. cholerae* is the causative biotype of the current cholera pandemic (1, 3). While the Classical biotype driving the former pandemics was characterized to exert its effects through CT, the El Tor biotype expresses the accessory toxins hemolysin and MARTX, both of which participate in host infection alongside CT (32). To determine the contribution of El Tor toxins to host innate defense mechanisms, we characterized the inflammasome activation pathways and their bacterial triggers during infection of murine macrophages with the N16961 El Tor *V. cholerae* strain.

*V. cholerae* is generally regarded as one of the Gram-negative enteropathogens that activates the non-canonical caspase-11-dependent inflammasome, owing to its capacity of CT-mediated LPS import in the cytosol. However, this prevailing notion stems from infecting mouse macrophages with the Classical biotype (20). In contrast, we report that the El Tor biotype of *V. cholerae* induces normal inflammasome responses in caspase-11-deficient murine macrophages. Furthermore, additional deletion of caspase-1 abolished El Tor *V. cholerae* induced IL-1β secretion, showing that this biotype triggers canonical inflammasome activation. Inflammasome responses induced by the El Tor biotype did not rely on CT expression. Indeed, the lack of CT expression in Δ*ctxAB* mutants did not affect inflammasome responses, while conversely boosting CT expression in the El Tor biotype through specific *in vitro* culturing methods did not augment inflammasome responses nor did it reveal a caspase-11 dependency.

Given these clear observations that the El Tor biotype *V. cholerae* induces a caspase-11- and CT-independent canonical inflammasome activating pathway, it is conceivable that the accessory toxins characteristic of this biotype are more efficient in activating the inflammasome than the CTB-mediated cytosolic delivery of LPS as observed in Classical *V. cholerae* strains. Consistent with this idea, infections using Δ*ctxAB/hlyA* mutants revealed a major contribution for the accessory toxin hemolysin to inflammasome activation by the El Tor biotype *V. cholerae*. Hemolysins are well-characterized pore-forming toxins and are the main lethal toxins of El Tor *V. cholerae* strains in murine studies (32, 33). Bacterial pore-forming toxins are known to cause efflux of intracellular K^+^ ions, a common signal sufficient for triggering Nlrp3-driven canonical inflammasome activation (34). Indeed, in agreement with identifying the pore-forming toxin hlyA as the bacterial instigator, genetic as well as pharmacological experiments identified host Nlrp3 as the main receptor responsible for initiating El Tor *V. cholerae* induced canonical inflammasome activation and subsequent IL-1β secretion.

Our observations showing that the El Tor *V. cholerae* N16961 strain triggers the Nlrp3 inflammasome align with a previous study showing that also the N86 El Tor strain activates the Nlrp3 inflammasome (35). Although this study used macrophages lacking both caspase-1 and caspase-11 and therefore could not discriminate between the canonical and non-canonical inflammasome pathways, their finding that also in the N86 strain hlyA was largely responsible for caspase-1 activation (35) suggests that the hemolysin-induced canonical Nlrp3 pathway observed in our study could be a general inflammasome activating mechanism among El Tor biotype strains. Interestingly, an hlyA-deficient El Tor N86 strain displayed residual inflammasome activation that could be abolished by additional deletion of the *rtxA* gene encoding the MARTX accessory toxin (35). Although this led the authors to speculate about mechanisms through which MARTX could potentially contribute to Nlrp3 inflammasome activation (35), our observations suggest that MARTX might instead trigger an Nlrp3-independent inflammasome. Indeed, similar to the hlyA-deficient N86 El Tor strain, we observed residual IL-1β maturation and secretion induced by Δ*ctxAB/hlyA* N16961 El Tor mutants. These inflammasome responses were lost in Pyrin^−/−^ macrophages, suggesting that the N16961 El Tor strain could trigger an Nlrp3-independent Pyrin inflammasome pathway. In line with this notion, Nlrp3 inhibition with MCC950 was only able to fully block *V. cholerae* induced IL-1β maturation and secretion in Pyrin-deficient conditions. These observations indicated that Pyrin activation constitutes an additional hlyA- and Nlrp3-independent driver of inflammasome responses to El Tor *V. cholerae* infection. Interestingly, the El Tor characteristic MARTX is capable of inactivating host Rho GTPases (36), which in the context of several other bacteria was shown to be the cytoskeletal damage induced event that activates the Pyrin inflammasome (19). Therefore, it is tempting to speculate that the observed Nlrp3- and hlyA-independent Pyrin inflammasome activation was induced by the MARTX El Tor accessory toxin. Such a pro-inflammatory role for the MARTX toxin would be in line with its known contribution to El Tor *V. cholerae* induced lethality as well as inflammation in mice (32, 37). Alternatively, a recent study showed that recombinant CTB can act as a Pyrin activator in LPS-stimulated murine peritoneal macrophages (38). However, since we show that a *V. cholerae* Δ*ctxAB/hlyA* mutant still activates Pyrin-dependent IL-1β secretion in murine BMDMs, CTB clearly is dispensable for Pyrin activation in bone marrow derived macrophages. This finding aligns with the observation that recombinant CTB activated the Pyrin inflammasome in peritoneal but not in bone marrow derived macrophages (38), and thus indicates that *V. cholerae* may engage different inflammasome activation mechanisms in a cell-type specific manner.

Our study demonstrating the importance of the differential toxin expression repertoire in Classical vs. El Tor *V. cholerae* biotypes for triggering inflammasome responses may explain previous observations in a human cell line. Indeed, although not pinpointing the activation of either canonical or non-canonical inflammasomes, a study using human THP-1 monocytes found that El Tor *V. cholerae* biotypes were more potent inflammasome activators when compared to Classical biotypes (13). However, in contrast to our observations revealing both hlyA-dependent and -independent modes of inflammasome activation in murine macrophages, IL-1β maturation in El Tor infected THP-1 cells depended entirely on hlyA (13), suggesting that El Tor *V. cholerae* does not activate the Pyrin inflammasome in the human THP-1 cell line. However, given the differential capacity of El Tor *V. cholerae* to trigger Pyrin inflammasome activation in bone marrow derived vs. peritoneal macrophages in mice (38), it remains possible that other human myeloid cell types are susceptible to this trait of the El Tor biotype.

Thus, our study shows that the El Tor *V. cholerae* N16961 strain utilizes distinct accessory toxins to drive caspase-11- and CT-independent canonical inflammasome activation through Nlrp3- and Pyrin-dependent mechanisms. While their relative contributions to pathology remain to be investigated, our study suggests that the El Tor *V. cholerae* biotype that drives the current seventh cholera pandemic (1, 3) may trigger parallel Nlrp3- and Pyrin-dependent pathways to induce secretion of inflammasome-dependent pro-inflammatory cytokines.

## Data Availability Statement

All datasets generated for this study are included in the article/supplementary material.

## Ethics Statement

The animal study was reviewed and approved by Ethical Committee Animal Experimentation—Ghent University—Faculty of Medicine and Health Sciences (permit number LA1400536).

## Author Contributions

MM and UF performed the experiments. MM, ML, and AW designed the experiments and analyzed the data. GL, ML, and AW supervised the project. MM, TA, and AW wrote the manuscript with input from all other authors.

### Conflict of Interest

ML is an employee of Janssen Pharmaceutica. The remaining authors declare that the research was conducted in the absence of any commercial or financial relationships that could be construed as a potential conflict of interest.
